# Clinicopathological characteristics and treatment outcomes of advanced SMARCA4‐deficient thoracic tumors

**DOI:** 10.1002/cam4.6809

**Published:** 2023-12-20

**Authors:** Anni Wang, Yueping Jin, Zhengqi Cao, Li Lu, Ziming Li

**Affiliations:** ^1^ Shanghai Lung Cancer Center, Shanghai Chest Hospital Shanghai Jiao Tong University, School of Medicine Shanghai China

**Keywords:** immunotherapy, SMARCA4, thoracic tumors, undifferentiated tumor

## Abstract

**Purpose:**

SMARCA4‐deficient thoracic tumors, characterized by distinct clinicopathological, morphological, immunohistochemical, and genetic features, differ significantly from conventional non‐small‐cell lung carcinomas (NSCLCs). This group encompasses both SMARCA4‐deficient NSCLCs (SMARCA4‐NSCLCs) and SMARCA4‐deficient undifferentiated tumors (SMARCA4‐UTs). The efficacy of PD‐1 inhibitors in treating SMARCA4‐deficient thoracic tumors remains uncertain.

**Methods:**

Medical records of 36 patients diagnosed with stage IIIB, IIIC, or IV SMARCA4‐deficient thoracic tumors were analyzed. We assessed the clinical, pathological, and genetic features of these patients through immunohistochemistry (IHC) and a 68‐gene panel next‐generation sequencing (NGS). We compared the differences between SMARCA4‐NSCLCs and SMARCA4‐UTs, and evaluated the impact of chemotherapy and immunotherapy on patient outcomes.

**Results:**

The majority of patients with SMARCA4‐deficient thoracic tumors were heavy‐smoking males, averaging 64.6 years in age. IHC predominantly showed weak or negative staining for markers such as TTF‐1, CK5/6, p40, synaptophysin, chromogranin A, and CD56, which are often associated with adenocarcinoma, squamous cell carcinoma, and neuroendocrine tumors. The most common genetic mutations identified via NGS included TP53, CDKN2A, KRAS, STK11, NF1, and PTEN. No significant overall survival (OS) difference was observed between SMARCA4‐NSCLCs and SMARCA4‐UTs (*p* = 0.366). The median OS for patients treated with chemotherapy (*n* = 9) was 447 days, while the median OS for patients undergoing PD‐1‐inhibitor‐based therapy (*n* = 16) was not reached (*p* = 0.105).

**Conclusion:**

SMARCA4‐deficient thoracic tumors exhibit distinct characteristics from conventional NSCLCs, and PD‐1 inhibitors show promise in treating advanced SMARCA4‐deficient thoracic tumors.

## 1 INTRODUCTION

The SWItch/Sucrose Non‐Fermenting (SWI/SNF) family is crucial in transcriptional regulation and DNA‐damage repair, and mutations of genes in SWI/SNF family were observed in about 25% of all cancers.^1^

As an ATPase subunit in mammalian SWI/SNF complex, SMARCA4 is believed to play a pivotal role in suppressing tumorigenesis in primary lung cancers.^2^, ^3^ Recent studies revealed frequent SMARCA4 mutations in various cancers, including rhabdoid tumors, small‐cell carcinomas of the ovary, hypercalcemic type (SCCOHTs), and non‐small‐cell lung cancers (NSCLCs).^3^ To date, 120 SMARCA4 alterations have been identified and approximately 41% are truncating mutations potentially leading to loss of expression and function of SMARCA4.^4^ Immunohistochemical studies showed that SMARCA4 protein expression deficiency is common in NSCLCs with SMARCA4 truncating mutations.^5^, ^6^ NSCLCs harboring mutated SMARCA4 are correlated with poorer outcomes and limited benefit from chemotherapy, but are demonstrated to be sensitive to immune checkpoint inhibitors.^5^, ^6^, ^7^ Patients with reduced or absent SMARCA4 expression experience shorter overall survival (OS).^8^, ^9^, ^10^ SMARCA4 deficiency impacts nucleosome positioning and induces accessibility at enhancers, which potentially contributes to NSCLC development and aggressiveness.^11^, ^12^, ^13^

In 2021, the World Health Organization (WHO) recognized SMARCA4‐deficient undifferentiated thoracic tumor (SMARCA4‐UT), formerly known as SMARCA4‐deficient thoracic sarcoma (SMARCA4‐DTS), as a distinct entity that was different from SMARCA4‐deficient NSCLC, due to its unique histological, immunohistochemical, clinical, and prognostic features.^14^ SMARCA4‐UT predominantly affects younger males with history of heavy smoking and presents as large masses often involving lungs and other thoracic structures.^15^, ^16^, ^17^, ^18^, ^19^ Histologically, these tumors are undifferentiated round cell or exhibit rhabdoid morphology.^20^ Transcriptome analyses aligned SMARCA4‐UTs more closely with SMARCA4‐mutated SCCOHTs and SMARCB1‐inactivated malignant rhabdoid tumors (MRTs) rather than conventional NSCLCs.^18^ Most cases reported to date are metastatic, exhibiting highly aggressive behavior and poor prognosis.^15^, ^16^, ^21^ However, the characteristics and clinical outcomes of advanced SMARCA4‐deficient thoracic tumors, particularly in response to immunotherapy, remain underexplored.

This study examines the clinical, pathological, immunohistochemical, and genetic profiles of 36 patients with advanced SMARCA4‐deficient thoracic tumors, focusing on their responses to chemotherapy and immunotherapy.

## 2 METHODS

### 2.1 Patients

This retrospective clinical study analyzed the medical records of patients with thoracic tumors diagnosed at Shanghai Chest Hospital from 2017 to 2022. Of these, 84 patients with SMARCA4‐deficient thoracic tumors were identified via immunohistochemistry (IHC) performed for diagnostic purpose. Patients in stage I, II, and IIIA (48 in total) were excluded, while 36 in stage IIIB, IIIC, and IV were included. Among them, 21 were diagnosed with SMARCA4‐NSCLC and 15 with SMARCA4‐UT from May 5, 2019 to August 12, 2022. Evaluated clinicopathological and genetic variables included sex, age at diagnosis, smoking history, primary tumor size, sites of distant metastases, TNM stage, surgical and radiotherapy treatments, first‐line and subsequent systematic treatment regimens, PD‐L1 tumor proportion score (PD‐L1 TPS), pathological and IHC results, and next‐generation sequencing (NGS) results. Staging was based on the eighth edition of AJCC TNM classification. Follow‐ups were conducted via medical records or telephone, with the last time on December 15, 2022. The institutional review board of Shanghai Chest Hospital approved this study.

### 2.2 Pathological analysis and IHC

Tissue samples were obtained through core needle biopsy, transbronchial needle aspiration (TBNA), and lobectomy for diagnostic purpose. Fresh tissue samples were fixed using 10% formalin, paraffin‐embedded, cut into 4 μm sections, and mounted on glass slides. Following deparaffinization and dehydration, hematoxylin and eosin (H&E) staining was applied. After antigen retrieval, immunohistochemical staining was performed utilizing antibodies against SMARCA4, p40, CK5/6, chromogranin A, CD56, TTF‐1, CK7, NapsinA, synaptophysin, NUT, SMARCB1, CD34, SALL4, HEP‐1, EMA, and vimentin (see Table S3 for details).

### 2.3 Next‐generation sequencing

Genomic profiling of 27 patients was conducted using a 68‐gene panel NGS. Fresh tumor tissue samples, formalin‐fixed and paraffin‐embedded, underwent DNA extraction using QIAamp DNA FFPE Tissue Kit (Qiagen, Hilden, Germany) following the manufacturer's instructions. A 68‐gene panel NGS was performed on the Nextseq500 sequencer (Illumina, Inc, Madison, WI, USA). The genomic profiles were characterized with Lung Core Panel (Buring Rock Biotech, Guangzhou, China). The tested genes are listed in Table S4.

### 2.4 Statistical analysis

Genetic characteristics were assessed using chi‐squared test. OS of SMARCA4‐NSCLCs and SMARCA4‐UTs was measured from the date of metastatic disease diagnosis to death. For estimating the efficacy of chemotherapy and PD‐1 inhibitors, OS was defined from first treatment to death. Data were censored when the event did not occur on the last follow‐up. Kaplan–Meier method was employed to estimate OS, and OS differences between groups were compared using log‐rank test. A *p* value <0.05 indicated statistical significance in all analyses. All data analyses were performed with IBM SPSS Statistics (version 22; IBM Corp, Armonk, NY, USA).

## 3 RESULTS

### 3.1 Clinicopathological characteristics

This study identified 36 patients with advanced SMARCA4‐deficient thoracic tumors using IHC during routine clinical practice. As outlined in Tables 1 and S1, a significant majority (*n* = 34, 94.4%) of the patients were male, with an average age of 64.6 years (range: 46–81 years). All 22 patients with documented smoking history were current or former smokers, averaging 42.6 pack‐years (range: 5–90). The majority had large primary tumors (mean size: 6.1 cm; range: 0.5–14.4 cm). Stage IV disease was predominant (*n* = 29, 80.6%), with bones (*n* = 14, 38.9%), adrenal glands (*n* = 8, 22.2%), brain (*n* = 6, 16.7%), and liver (*n* = 3, 8.3%) being the most common metastatic sites. Histologically, 21 patients (58.3%) were diagnosed with SMARCA4‐NSCLCs, which included 15 NSCLCs not otherwise specified (41.7%), 4 adenocarcinomas (11.1%), 1 squamous cell carcinoma (2.8%) and 1 adenosquamous carcinoma (2.8%). The remaining 15 patients (41.7%) had SMARCA4‐UTs. Only a small proportion (*n* = 3, 10.7%) showed high PD‐L1 expression, whereas the majority (60.7%, *n* = 17) of tested patients were PD‐L1 negative.

**TABLE 1:** The clinicopathological characteristics of patients with SMARCA4‐deficient thoracic tumors.

Characteristics	*n* = 36
Sex, *n* (%)	
Male	34 (94.4%)
Female	2 (5.6%)
Age at diagnosis, mean (range)	64.6 ± 8.79 (46–81)
TNM stage, *n* (%)	
III B	4 (11.1%)
III C	3 (8.3%)
IV	29 (80.6%)
Smoking history	
Current or former smoker	22 (61.1%)
N.A.	14 (38.9%)
Smoking pack‐years, mean (range)	42.6 ± 24.16 (5–90)
Primary tumor size (cm), mean (range)	6.1 ± 3.60 (0.5–14.4)
Distant metastasis, *n* (%)	
Bones	14 (38.9%)
Adrenal glands	8 (22.2%)
Brain	6 (16.7%)
Liver	3 (8.3%)
Histology, *n* (%)	
SMARCA4‐NSCLC	21 (58.3%)
NSCLC, NOS	15 (41.7%)
Adenocarcinoma	4 (11.1%)
Squamous cell carcinoma	1 (2.8%)
Adenosquamous carcinoma	1 (2.8%)
SMARCA4‐UT	15 (41.7%)
PD‐L1 TPS, *n* (%)	
<1%	17 (60.7%)
1%–50%	8 (28.6%)
>50%	3 (10.7%)
N.A.	8 (28.6%)

Abbreviations: N.A., not available; NOS, not otherwise specified; PD‐L1 TPS, PD‐L1 tumor proportion score; SMARCA4‐NSCLC, SMARCA4‐deficient non‐small‐cell lung carcinoma; SMARCA4‐UT, SMARCA4‐deficient undifferentiated tumor.

### 3.2 Pathological and immunohistochemical characteristics

**FIGURE 1:**
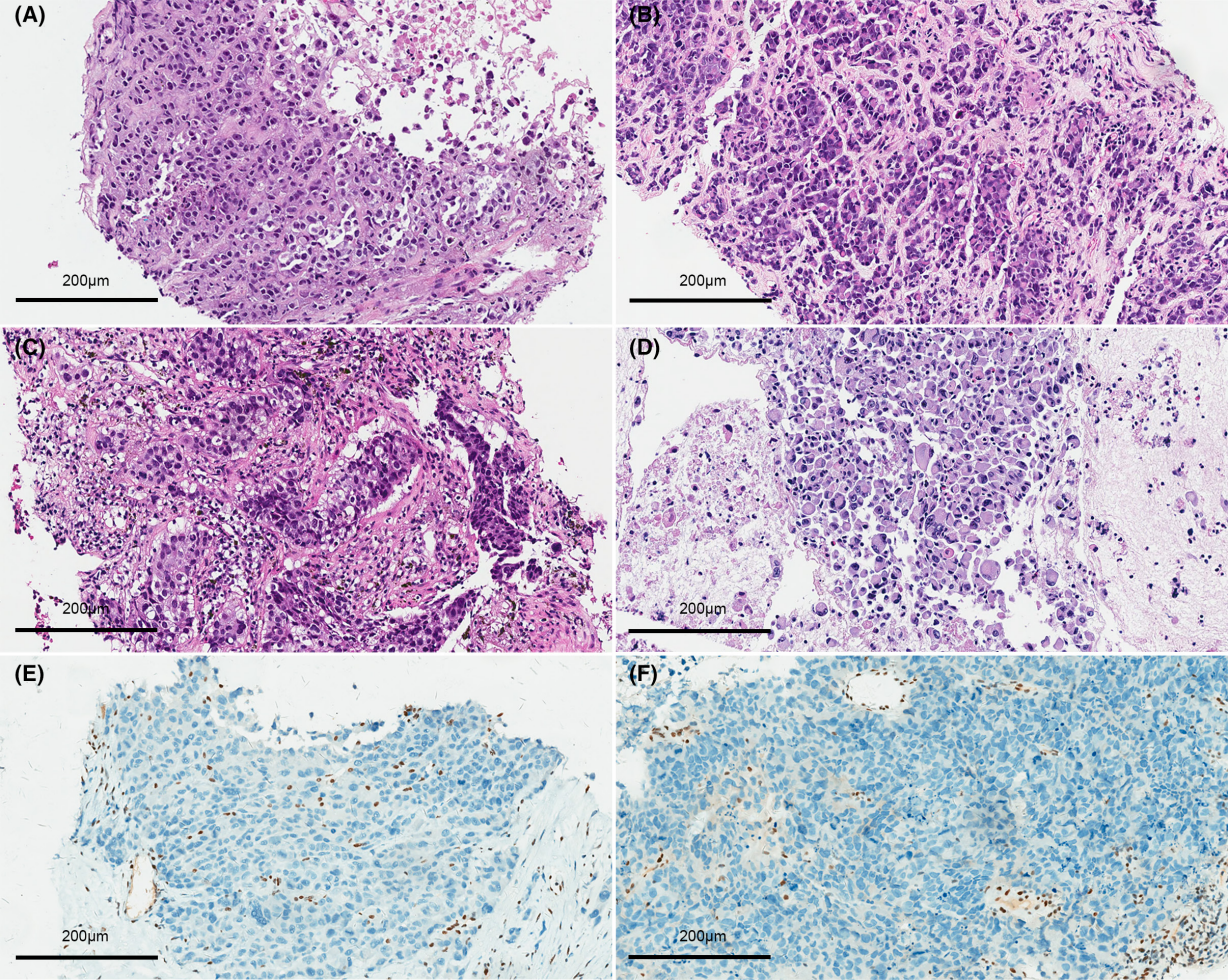
Representative histological results of SMARCA4‐deficient thoracic tumors. (A) In SMARCA4‐UT cases, tumor cells are discohesive undifferentiated large round cells with prominent nuclei and nucleoli. (B) Typical rhabdoid cells with hyaline eosinophilic inclusions in SMARCA4‐UTs. (C) SMARCA4‐NSCLC tumor cells with cohesion and pleomorphism arranged in nests. (D) Multinucleation in SMARCA4‐NSCLC tumor cells. (E, F) Immunohistochemical staining shows loss of SMARCA4 expression in (E) SMARCA4‐UT and (F) SMARCA4‐NSCLC cases. As internal positive control, the background cells retain SMARCA4 expression. SMARCA4‐NSCLC, SMARCA4‐deficient non‐small‐cell lung carcinoma; SMARCA4‐UT, SMARCA4‐deficient undifferentiated tumor.

Specimens were obtained from metastatic lesions in supraclavicular lymph nodes (4 of 36, 11.1%) and primary tumors (24 of 36, 66.7%) using core needle biopsy (28 of 36, 77.8%), TBNA (6 of 36, 16.7%), and lobectomy (2 of 36, 5.6%). In most SMARCA4‐UT cases, tumor cells typically appeared as monomorphic, undifferentiated round or oval cells with large nuclei **(**Figure 1A,B **)** and occasionally prominent nucleoli, exhibiting discohesive arrangement. Some cases showed rhabdoid cells characterized by large cells with eosinophilic hyaline inclusions in the cytoplasm **(**Figure 1B **)**. SMARCA4‐NSCLC tumor cells were more cohesive, displaying from mild to moderate pleomorphism and clear or pale eosinophilic cytoplasm **(**Figure 1C,D **)**. SMARCA4‐NSCLC tumor cells with two or even multiple nuclei were observed occasionally **(**Figure 1D **)**. In several cases, SMARCA4‐NSCLC tumor cells presented nested pattern **(**Figure 1C **)**. Extensive necrosis was frequently observed in both types **(**Figure 1A,D **)**.

The immunohistochemical characteristics are detailed in Table S2. All 36 patients lacked SMARCA4 expression, and representative cases of SMARCA4‐UTs and SMARCA4‐NSCLCs are shown in Figure 1E,F. SMARCA4‐NSCLCs and SMARCA4‐UTs showed similar immunohistochemical features. The majority were negative for markers including p40 (88.6%), CK5/6 (88.9%), chromogranin A (91.7%), and CD56 (96.9%). TTF‐1, CK7, and NapsinA were positive in 17 (50%), 14 (58.3%), and 2 patients (6.06%), respectively. Synaptophysin positivity was found in 12 cases (70.6%). Ki67 proliferation rate averaged 52% (*n* = 19, range: 20%–70%). All tested cases were consistently negative for NUT (*n* = 15) and positive for SMARCB1 (*n* = 25). CD34 and SALL4 were positive in three of fourteen (21.4%) and two of four cases (50%). HEP‐1 was expressed in two of four cases (50%). Two tested SMARCA4‐NSCLCs were negative for EMA. Of note, tumor cells in 12 cases (80%) with SMARCA4‐UTs expressed vimentin, whereas only 5 cases (41.7%) with SMARCA4‐NSCLCs were positive for vimentin.

### 3.3 Genetic characteristics

**FIGURE 2:**
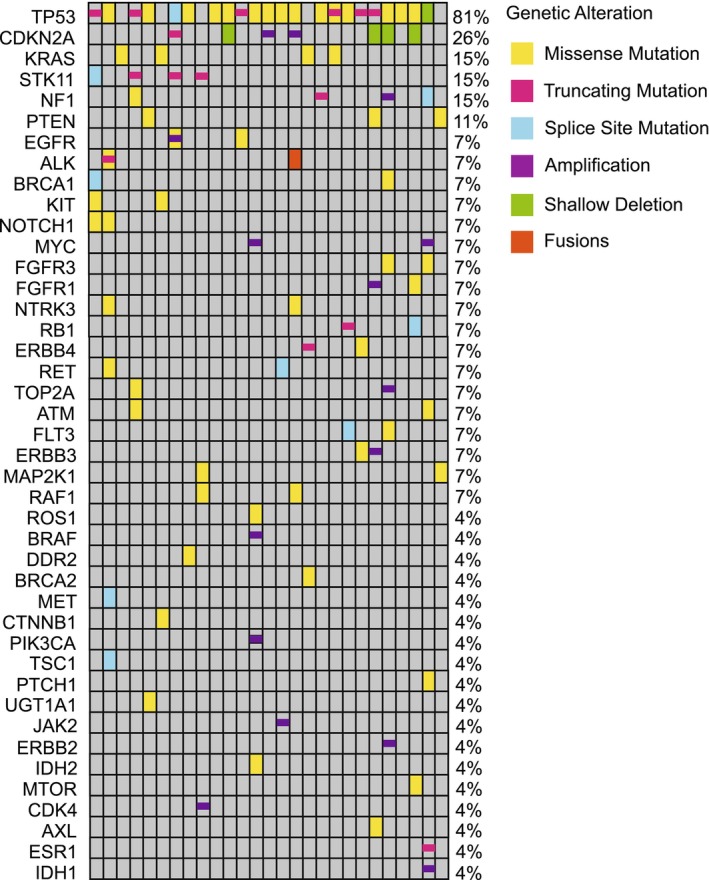
Landscape of mutated genes of cases with SMARCA4‐deficient thoracic tumors. Twenty‐seven cases with SMARCA4‐deficient thoracic tumors were analyzed with a 68‐gene panel next‐generation sequencing.

We evaluated the genetic characteristics of 27 cases (Figure 2) using a 68‐gene panel NGS, excluding 9 cases, owing to lack of NGS results. Among all the tested cases with SMARCA4‐deficient thoracic tumors, TP53 was the most frequently mutated gene (81%), followed by CDKN2A (26%), KRAS (15%), STK11 (15%), NF1 (15%), and PTEN (11%). As shown in Table 2, the mutational spectrum of SMARCA4‐NSCLCs and SMARCA4‐UTs was similar. TP53, STK11, NF1, and KRAS were the most commonly mutated genes of SMARCA4‐NSCLCs, and most frequently observed co‐mutated genes of SMARCA4‐UTs were TP53 and CDKN2A.

**TABLE 2:** Mutated genes in SMARCA4‐NSCLC and SMARCA4‐UT cases.

Genes	SMARCA4‐NSCLC (*n* = 15)	SMARCA4‐UT (*n* = 12)	*p*
TP53	11 (73.3%)	11 (91.7%)	0.223
STK11	4 (26.7%)	0	0.053
NF1	3 (20.0%)	1 (8.3%)	0.396
CDKN2A	2 (13.3%)	5 (41.7%)	0.095
KRAS	3 (20.0%)	1 (8.3%)	0.396

Abbreviations: SMARCA4‐NSCLC, SMARCA4‐deficient non‐small‐cell lung carcinoma; SMARCA4‐UT, SMARCA4‐deficient undifferentiated tumor.

### 3.4 Clinical outcome of SMARCA‐NSCLCs and SMARCA4‐UTs

**FIGURE 3:**
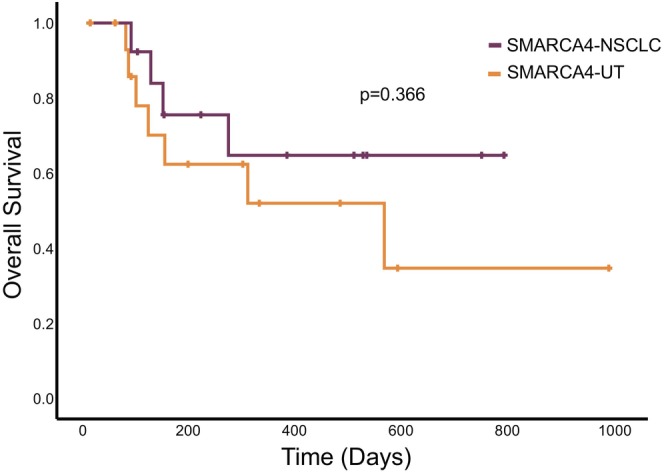
Comparison of overall survival for patients with advanced SMARCA4‐deficient non‐small‐cell lung carcinomas (SMARCA4‐NSCLCs) and SMARCA4‐deficient undifferentiated tumors (SMARCA4‐UTs).

We assessed the clinical outcomes of SMARCA4‐NSCLCs and SMARCA4‐UTs, excluding 7 of 36 patients (19%) from the analysis due to loss to follow‐up after diagnosis. The groups consisted of 16 patients with SMARCA4‐NSCLCs and 13 with SMARCA4‐UTs. By the end of the follow‐up period, seven patients (44%) with SMARCA4‐NSCLCs and four of fourteen (31%) with SMARCA4‐UTs had died. The median OS for SMARCA4‐NSCLCs was 560 days [95% CI: 58–1062 days], while the median OS for SMARCA4‐UTs was not reached. There was no significant difference in OS between the two groups (*p* = 0.366) **(**Figure 3 **)**.

### 3.5 Outcomes for chemotherapy and PD‐1‐inhibitor‐based therapy

Our evaluation of the outcomes for platinum‐based chemotherapy and PD‐1‐inhibitor‐based therapy revealed that, among the nine patients who received chemotherapy without PD‐1 inhibitors, six (67%) died, one (11%) survived, and two (22%) were lost to follow‐up. Two patients received anlotinib as second‐line treatment. One patient harboring EML4‐ALK rearrangement was initially treated with alectinib and experienced progressive disease (PD) after 4 months. Afterward, this patient underwent one course second‐line chemotherapy before death. Regarding immunotherapy, eight of sixteen patients (50%) were alive at the time of data cutoff, and four (25%) had died. Ten patients received first‐line immunotherapy combined with platinum doublet chemotherapy, two patients received first‐line immunotherapy combined with anlotinib, and four patients were administered PD‐1 inhibitors with chemotherapy as second‐ or later‐line therapy.

**FIGURE 4:**
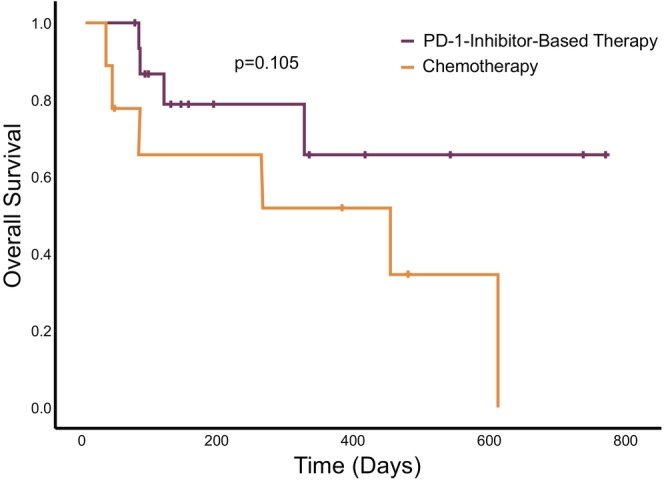
Survival analysis for overall survival on PD‐1‐inhibitor‐based therapy and chemotherapy.

Figure 4 displays the survival curves for patients treated with or without PD‐1 inhibitors. The median OS for patients not receiving PD‐1 inhibitors was 447 days (95% CI: 11–883 days). In contrast, the median OS for those undergoing immunotherapy was not reached. However, the difference in OS between PD‐1‐inhibitor‐based therapy and chemotherapy for advanced SMARCA4‐deficient thoracic tumors was not statistically significant (*p* = 0.105).

## 4 DISCUSSION

In 2015, Loarer et al. identified SMARCA4‐DTS as a distinct entity, which is characterized by unique transcriptomic profiles and immunohistochemical features.^18^ Further research elucidated the clinicopathological and genetic characteristics of SMARCA4‐DTS, as well as its response to therapeutic interventions.^15^, ^16^, ^19^, ^20^, ^22^ Renamed as “SMARCA4‐deficient undifferentiated tumor,” this tumor type distinctly differs from SMARCA4‐deficient NSCLC in phenotype.^14^ Subsequent studies described SMARAC4‐UTs as highly aggressive tumors that predominantly occur in male patients with younger age and heavy smoking history.^17^, ^23^, ^24^ Despite this, the response of SMARCA4‐UTs to PD‐1 inhibitors remains inconsistently reported and underexplored.^23^, ^24^ In this study, we evaluated the clinicopathological, immunohistochemical, and genetic characteristics of 36 patients with advanced SMARCA4‐deficient thoracic tumors, consisting of 21 SMARCA4‐NSCLCs and 15 SMARAC4‐UTs, and analyzed responses to platinum‐based chemotherapy and PD‐1 inhibitors.

This study included 36 patients with stage IIIB (*n* = 4), IIIC (*n* = 3), or IV (*n* = 29) disease, who were predominantly male (94.4%), with an average age of 64.6 years (range 46–81 years). Notably, three patients were diagnosed before the age of 50 years. All patients with available smoking history (*n* = 22) were current or former smokers and most of them were heavy smokers (*n* = 19). Most patients had large primary tumors (mean size = 6.1 cm; range: 0.5–14.4 cm). Clinically, SMARCA4‐NSCLCs and SMARCA4‐UTs shared similar features and high‐grade malignancy.^16^, ^25^, ^26^

Immunohistochemical analysis revealed loss of SMARCA4 expression in all 36 cases. Most immunohistochemical features of SMARCA4‐NSCLCs and SMARCA4‐UTs exhibited significant similarity. Consistent with previous studies, most cases with SMARCA4‐NSCLCs and SMARCA4‐UTs were negative for squamous cell carcinoma and neuroendocrine markers (CK5/6, p40, chromogranin A, and CD56), and the vast majority of SMARCA4‐UTs (60%) were negative for adenocarcinoma marker TTF‐1, reinforcing their distinction from conventional NSCLC.^19^, ^25^, ^26^ All cases were NUT negative, in accordance with the findings of Perret et al.^19^ Cytokeratin 7 was predominantly positive in SMARCA4‐NSCLCs (92.3%), but focal weak positivity of Cytokeratin 7 was only observed in two of nine (22.2%) SMARCA4‐UTs.^25^, ^26^, ^27^ Meanwhile, similar to the findings of Rekhtman et al., vimentin expression was more common in SMARCA4‐UTs (80%), compared to five of twelve cases (41.7%) in SMARCA4‐NSCLCs.^16^ All tested 25 cases expressed SMARCB1, highlighting distinct mutation patterns in subunits of SWI/SNF complexes.^1^ As reported by previous studies, more than 90% cases of SCCOHT harbored inactivating mutations of SMARCA4, which was perceived as a critical biomarker for diagnosis.^28^ Indeed, these studies indicated that SMARCA4 drove tumorigenesis in SCCOHT.^28^, ^29^ Conversely, while nearly all cases of extracranial rhabdoid tumors harbored mutations of SMARCB1, SMARCA4 mutations were only observed in approximately 0.5%–2% atypical teratoid/rhabdoid tumors.^30^, ^31^

Our NGS analysis of 27 SMARCA4‐deficient thoracic tumors identified frequent mutations in TP53, CDKN2A, KRAS, STK11, NF1, and PTEN, with rare actionable oncogenic driver mutations.^6^, ^17^ Two cases harbored EGFR L858R mutation, and both were lost to follow‐up after diagnosis. One patient harboring EML4‐ALK rearrangement received first‐line alectinib and experienced PD after 4 months. The absence of actionable mutations limited available treatment options and potential benefit of tyrosine kinase inhibitors for patients with SMARCA4‐deficient thoracic tumors.

The median OS for patients undergoing immunotherapy was not reached, whereas that was 447 days for those without immunotherapy (95% CI: 11–883 days). Of patients treated with PD‐1‐inhibitor‐based therapy, eight survived at data cutoff, compared to only one survivor among those receiving chemotherapy alone. However, due to the small cohort size and short follow‐up duration, these differences were not statistically significant.

Recent studies have introduced innovative therapeutic strategies. Given that SMARCA4 and SMARCA2 are complementary roles, Oike et al. confirmed a synthetic lethal relationship between them, where downregulation of SMARCA2 hindered the growth of SMARCA4‐deficient cancer cells.^32^ Furthermore, cells harboring SMARCA4 mutation exhibited enhanced oxygen consumption and respiratory capacity, displaying pronounced sensitivity to oxidative phosphorylation inhibitor IACS‐010759.^33^ In addition, these cells were demonstrated to have susceptibility to ATR and Aurora kinase A inhibitors.^34^, ^35^ Despite the typically poor prognosis for patients with SMARCA4‐deficient thoracic tumors, these findings offer promising avenues for treatment.

This study, centering on advanced SMARCA4‐deficient thoracic tumors, sought to delineate their clinical, pathological, immunohistochemical, and genetic attributes. It also underscored the efficacy of immunotherapy in these tumors. Nevertheless, this study faces several constraints. The sample size was limited, which necessitates further research for accurate assessments of clinicopathological and molecular characteristics and efficacy of immunotherapy. Limitations in the routine diagnostic approaches, including IHC and NGS, led to incomplete molecular data and PD‐L1 TPS in some patients, along with the absence of certain immunohistochemical markers due to insufficient tissue samples. The relatively short duration of follow‐up for patients undergoing systematic therapy may have contributed to the lack of statistically significant differences in OS. Moreover, the 68‐gene NGS panel did not encompass SMARCA4, which omitted cases with SMARCA4 mutations but intact protein expression. Future research should investigate the links between SMARCA4 mutations, non‐mutational causes of protein deficiency, and protein expression deficiency, utilizing both NGS and IHC. The limited NGS panel potentially restricted a comprehensive understanding of the genetic features of SMARCA4‐deficient thoracic tumors.

## 5 CONCLUSION

This study confirms that patients with advanced SMARCA4‐deficient thoracic tumors are predominantly male, with mean age of 64.6 years and history of heavy smoking. Mutations in TP53, CDKN2A, KRAS, STK11, NF1, and PTEN are common in these patients. PD‐1 inhibitors showed promising efficacy in treating advanced SMARCA4‐deficient thoracic tumors.

## AUTHOR CONTRIBUTIONS

**Anni Wang:** Conceptualization (lead); data curation (equal); formal analysis (equal); writing – original draft (lead). **Yueping Jin:** Conceptualization (lead); data curation (equal); formal analysis (equal); writing – review and editing (lead). **Zhengqi Cao:** Data curation (equal); formal analysis (equal). **Li Lu:** Data curation (equal); formal analysis (equal). **Ziming Li:** Conceptualization (lead); funding acquisition (lead); project administration (lead); resources (lead); writing – review and editing (lead).

## FUNDING INFORMATION

This study was funded by the National Natural Science Foundation of China (82072564), the Program of Shanghai Academic Research Leader (22XD142280), Shanghai Municipal Health Commission (2022XD029), the Innovative Research Team of High‐level Local Universities in Shanghai (SHSMU‐ZLCX20212302), Lian Yun Gang Shi Hui Lan Public Foundation (HL‐HS2020‐65), and National Multi‐disciplinary Treatment Project for Major Diseases (2020NMDTP).

## CONFLICT OF INTEREST STATEMENT

The authors declare no conflicts of interest.

## ETHICS STATEMENT

Written informed consent was obtained from all patients in this study. This study was approved by the institutional review board of Shanghai Chest Hospital.

## Supporting information


Data S1.

## Data Availability

The authors confirm that the data supporting findings of this research are available within the article and the supplementary tables.
